# A Case of Pulmonary Arteriovenous Shunt Diagnosed by Microbubble Test via a Swan-Ganz Catheter

**DOI:** 10.7759/cureus.75794

**Published:** 2024-12-16

**Authors:** Keiji Hoshino, Koji Kurosawa, Nogiku Niwamae, Munenori Ide, Shoichi Tange

**Affiliations:** 1 Department of Cardiology, Japanese Red Cross Maebashi Hospital, Maebashi, JPN; 2 Department of Clinical Laboratory, Japanese Red Cross Maebashi Hospital, Maebashi, JPN; 3 Department of Pathology, Japanese Red Cross Maebashi Hospital, Maebashi, JPN

**Keywords:** hepatopulmonary syndrome, hypoxemia, microbubble test, pulmonary arteriovenous shunt, swan-ganz catheter

## Abstract

When encountering severe hypoxemia that does not respond to oxygen supplementation, it is essential to consider underlying right-to-left shunting. Among various diagnostic approaches, the microbubble test via transthoracic echocardiography (TTE) is a simple, noninvasive method for detecting pulmonary arteriovenous shunts, particularly in hepatopulmonary syndrome (HPS). Although microbubbles are usually administered peripherally, using a Swan-Ganz (SG) catheter to inject microbubbles directly into the pulmonary artery may provide even more definitive diagnostic information. We report a case of a woman in her 60s who was admitted in cardiac arrest after several months of progressive dyspnea and one month of poor appetite. While resuscitation was successful, severe hypoxemia persisted despite intubation, mechanical ventilation, and oxygen therapy. Immediately after resuscitation, TTE revealed no intracardiac shunt but did show microbubbles in both heart chambers. The patient exhibited refractory hypoxemia, high-output status, and pulmonary hypertension. Based on a history of severe dietary irregularities, beriberi heart disease was suspected, and empirical thiamine administration improved pulmonary hypertension and high-output state; however, the hypoxemia remained unresolved. Since microbubbles had been observed in the left heart without an intracardiac shunt, a pulmonary arteriovenous shunt was suspected. Peripheral microbubble testing showed Grade 4 opacification of the left heart three to four heartbeats subsequent to the filling of the right heart. Concerns regarding hemodynamic instability and timing prompted a microbubble test via the already-placed SG catheter. Injection of microbubbles directly into the pulmonary artery again demonstrated Grade 4 opacification in the left heart alone, confirming a pulmonary arteriovenous shunt independent of intracardiac pathways. The patient later died on day six due to irreversible brain damage. The autopsy revealed cirrhosis with histopathological features of autoimmune hepatitis and pulmonary changes suggestive of a pulmonary arteriovenous shunt. Postmortem evaluations indicated low vitamin B1 levels, consistent with beriberi heart disease, and the presence of autoimmune markers suggesting Sjögren's syndrome. In beriberi heart disease, high output is associated with reduced vascular resistance and increased metabolic demand. In liver disease, excessive nitric oxide and carbon monoxide production may worsen these hemodynamic conditions, promoting pulmonary vasodilation and pulmonary arteriovenous shunt formation. Using the SG catheter for the microbubble test proved practical and effective, especially when standard peripheral methods are complicated by hemodynamic instability, difficulty visualizing the right heart, the potential presence of intracardiac shunts, or contraindications to transesophageal echocardiography (TEE). This technique, relying on commonly available cardiovascular equipment, may facilitate earlier diagnosis of shunt-related diseases in complex clinical scenarios.

## Introduction

When patients present with severe hypoxemia resistant to oxygen supplementation and positive pressure ventilation, the differential diagnosis should include conditions associated with right-to-left shunting. This encompasses both intracardiac and intrapulmonary shunts, of which the latter can occur in hepatopulmonary syndrome (HPS), a complication of advanced liver disease [1]. Patients with liver disease frequently develop HPS due to pulmonary neovascularization and excessive vasodilation [2,3]. The transthoracic echocardiography (TTE)-based microbubble test is a simple, noninvasive, and highly useful tool for detecting pulmonary arteriovenous shunts in HPS [4]. However, peripheral microbubble administration may be less reliable if hemodynamic instability exists, if the right heart is difficult to visualize, or if intracardiac shunts cannot be fully excluded [5,6]. In such complex scenarios, administering microbubbles directly into the pulmonary artery through a Swan-Ganz (SG) catheter offers a practical and effective diagnostic method that is unaffected by these challenging conditions.

Here, we report a case in which a pulmonary arteriovenous shunt was diagnosed using a microbubble test via an SG catheter in a patient who presented in cardiac arrest with unstable hemodynamics due to beriberi heart disease and who later was found to have autoimmune hepatitis (AIH)-induced cirrhosis and HPS.

This case was presented as an oral presentation at the 32nd Annual Scientific Meeting of the Japanese Society of Echocardiography, held from April 23 to 25, 2021, and received the Best-Case Presentation Award.

## Case presentation

A woman in her 60s was brought to our hospital in a state of cardiac arrest. For several months, her dyspnea had progressively worsened, and for the past month, she experienced loss of appetite. She had not sought medical attention during this time. Although she had a history of hypertension, she had discontinued her treatment on her own. On the day of admission, a colleague found her foaming at the mouth and unable to move, and an emergency call was placed.

Following resuscitation, the patient was admitted to the intensive care unit, but hypoxemia persisted despite mechanical ventilation. Thoracentesis to remove pleural fluid and bronchoscopy to remove sputum were performed, but both were ineffective. Coronary angiography and SG catheter placement were performed to evaluate ischemic heart disease, persistent hypoxemia, and high-output state after cardiac arrest. No coronary artery lesions were found, but postcapillary pulmonary hypertension was noted with a mean pulmonary artery wedge pressure (PAWP) of 25 mmHg and a mean pulmonary artery pressure of 29 mmHg. The Fick method calculated cardiac output (CO) at 8.16 L/minute and cardiac index (CI) at 5.40 L/minute/m^2^, with low pulmonary vascular resistance (PVR) of 0.49 WU and systemic vascular resistance (SVR) of 10.4 WU. Sampling showed no O_2_ step-up.

A comparison with standard hemodynamic values further supported the diagnosis. The mean PAWP exceeded the normal range of 8-15 mmHg, consistent with postcapillary pulmonary hypertension. PVR was markedly reduced compared to the normal upper limit of approximately 3 WU, while SVR remained within the lower end of the normal range (10-15 WU). CO and CI were significantly elevated beyond their respective normal ranges of 4-8 L/minute and 2.2-4.0 L/minute/m^2^, supporting the presence of a high-output state [7].

These findings confirmed a diagnosis of high-output and postcapillary pulmonary hypertension characterized by decreased SVR and PVR. From the initial admission evaluations, no common causes of a high-output state, such as hyperthyroidism, sepsis, or anemia, were identified. A detailed history from the family revealed significant dietary irregularities, raising suspicion of beriberi heart disease. Based on this suspicion and without waiting for the vitamin B1 level results, thiamine was administered as a diagnostic therapy. This thiamine administration confirmed the diagnosis of beriberi heart disease by alleviating the pulmonary hypertension and high output. Specifically, it rapidly reduced the mean pulmonary artery pressure from 29 to 20 mmHg, CI from 5.40 to 3.40 L/minute/m^2^, and increased SVR from 10.4 to 18.1 WU. Although an attempt was made to re-measure PAWP, unfortunately, a stable wedge position could no longer be achieved. Despite hemodynamic normalization, hypoxemia persisted. Given the appearance of microbubbles in the left heart on TTE, diseases causing right-to-left shunting were suspected as the cause of hypoxemia.

**Figure 1:**
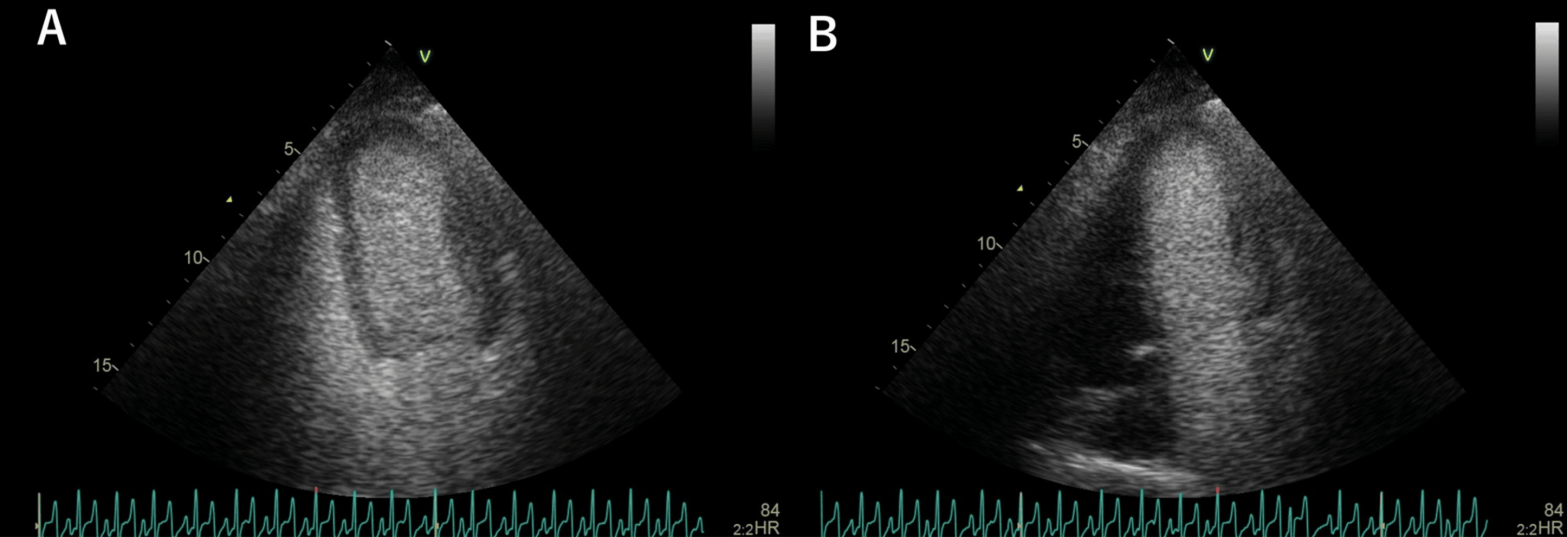
Microbubble test A: Peripheral route microbubble test showing Grade 4 microbubble opacification in the left heart three to four beats after the right heart. B: Microbubble test via the distal lumen of the SG catheter placed in the pulmonary artery, showing Grade 4 microbubble opacification exclusively in the left heart.

A peripheral microbubble test showed Grade 4 microbubble opacification of the left heart three to four heartbeats after right heart filling (Figure 1A) (Video 1). Although a pulmonary arteriovenous shunt was initially suspected, there were concerns regarding the presence of beriberi heart disease and the potential for hemodynamic instability since the acute post-cardiac arrest period. As with standard diagnostic criteria, relying solely on the timing of microbubble appearance in the left heart to distinguish between intracardiac and intrapulmonary shunts was considered uncertain. Moreover, due to the patient's prolonged hypoxemia, a TEE-based evaluation for ruling out intracardiac shunts was deemed risky.

**Video 1:** Microbubble test from the peripheral route

**Figure 2:**
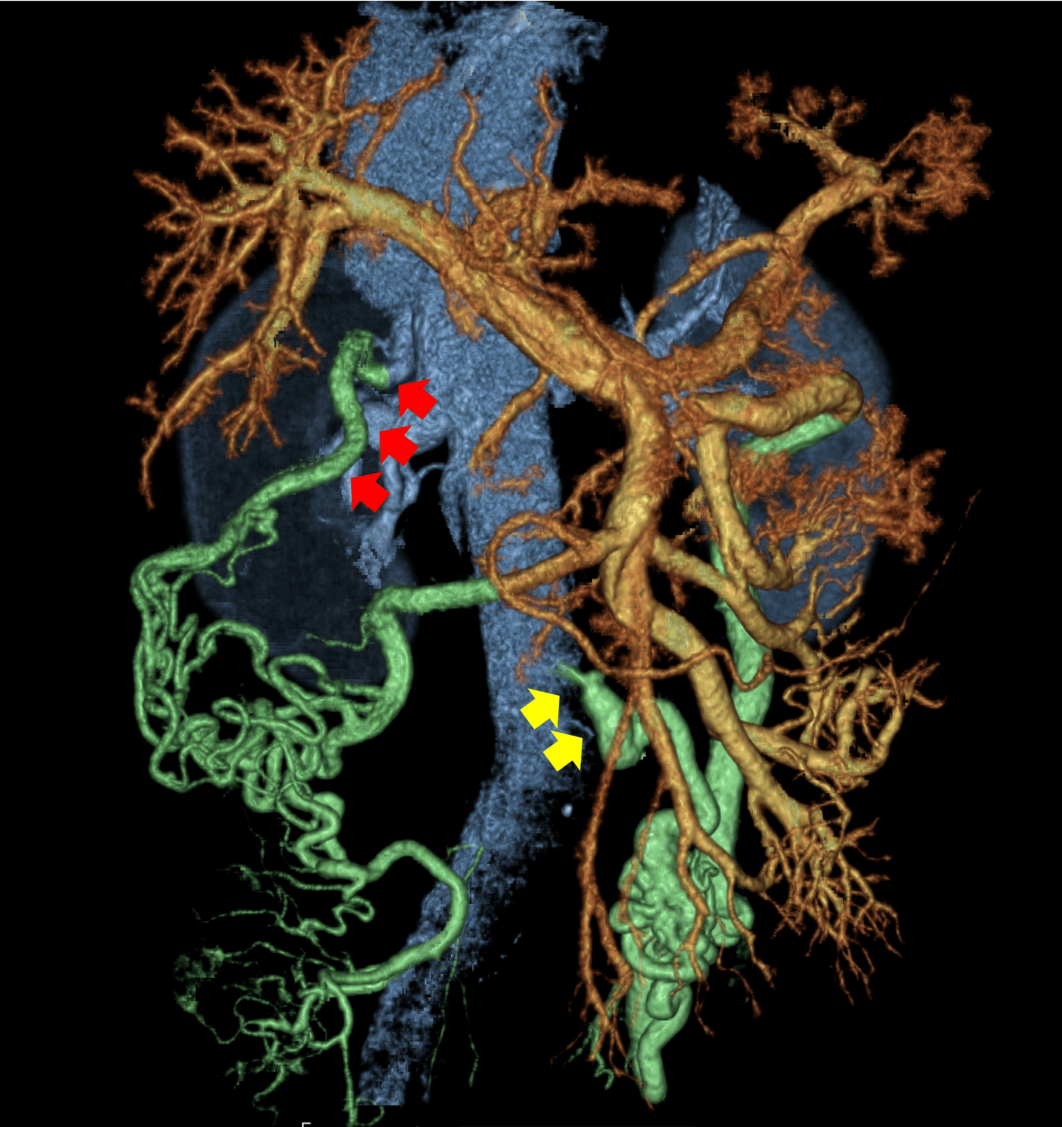
Contrast-enhanced CT Contrast CT showing abnormal blood vessels draining from the portal system into the inferior vena cava (yellow arrows) and the right renal vein (red arrows), indicating the formation of a portosystemic shunt.

Therefore, additional evaluation was performed by injecting microbubbles directly into the pulmonary artery through the already-placed SG catheter after confirming via pressure waveforms that the catheter tip was not wedged. This approach effectively eliminated potential interference from intracardiac shunts. Following standard peripheral microbubble testing protocols [8], a mixture of 8 mL saline, 1 mL air, and 1 mL patient blood was agitated to generate microbubbles, which were subsequently injected through the distal lumen of the SG catheter. Grade 4 opacification of innumerable microbubbles was observed exclusively in the left heart (Figure 1B, Video 2), conclusively confirming a pulmonary arteriovenous shunt without intracardiac involvement. Although contrast-enhanced CT revealed no significant abnormal pulmonary vessels suggesting a shunt, it did show abnormal vessels returning from the portal system to the inferior vena cava and right renal vein, indicating a portosystemic shunt (Figure 2).

**Video 2:** Microbubble test from the distal lumen of the SG catheter placed in the pulmonary artery

Despite systemic management, her consciousness did not recover, and a head CT scan revealed severe brain damage. After discussions with the family, who declined further aggressive treatments, palliative care was instituted. She died on day six.

**Figure 3:**
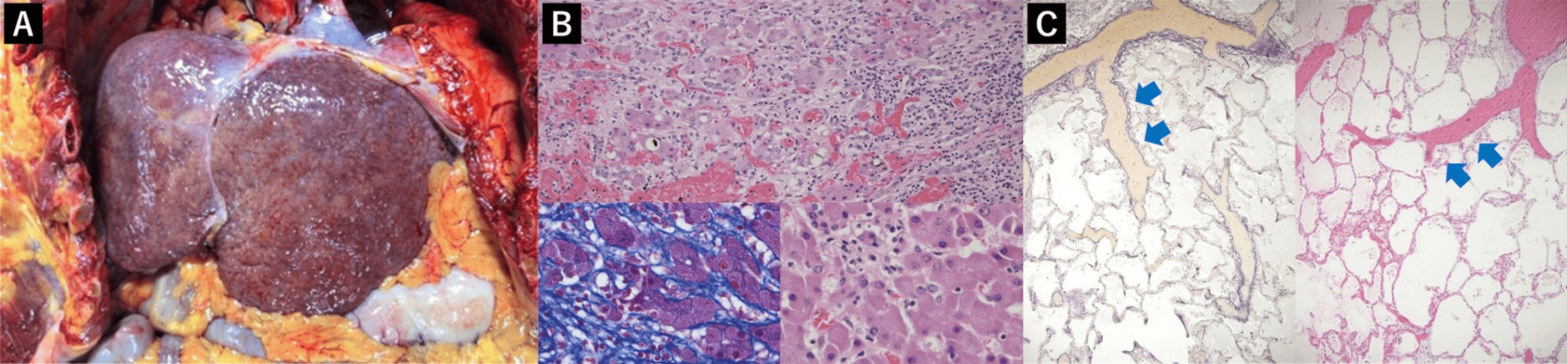
Pathological findings A: Macroscopic view showing cirrhosis. B: Liver pathology showing lymphocyte infiltration in the portal area, fibrous bridging, and disrupted lobular architecture. Emperipolesis, where lymphocytes invade the cytoplasm of hepatocytes, indicative of autoimmune hepatitis, is observed (left bottom: Masson's trichrome staining, ×400; others: hematoxylin and eosin staining, top: ×200, right bottom: ×400). C: In the lung tissue, the pulmonary artery is peripherally dilated, suggesting a pulmonary arteriovenous shunt (blue arrows) (left: Elastica van Gieson staining; right: hematoxylin and eosin staining, ×12.5).

The autopsy revealed cirrhosis with lymphocyte infiltration, emperipolesis (lymphocytes invading the hepatocyte cytoplasm), fibrous bridging, and disrupted lobular architecture, all consistent with AIH (Figures 3A, 3B). Emperipolesis is characteristic of AIH [9]. The lungs showed peripheral expansion of pulmonary arteries, suggesting pulmonary arteriovenous shunt formation (Figure 3C) [10]. There were no intracardiac shunts or structural heart disease identified.

**Figure 4:**
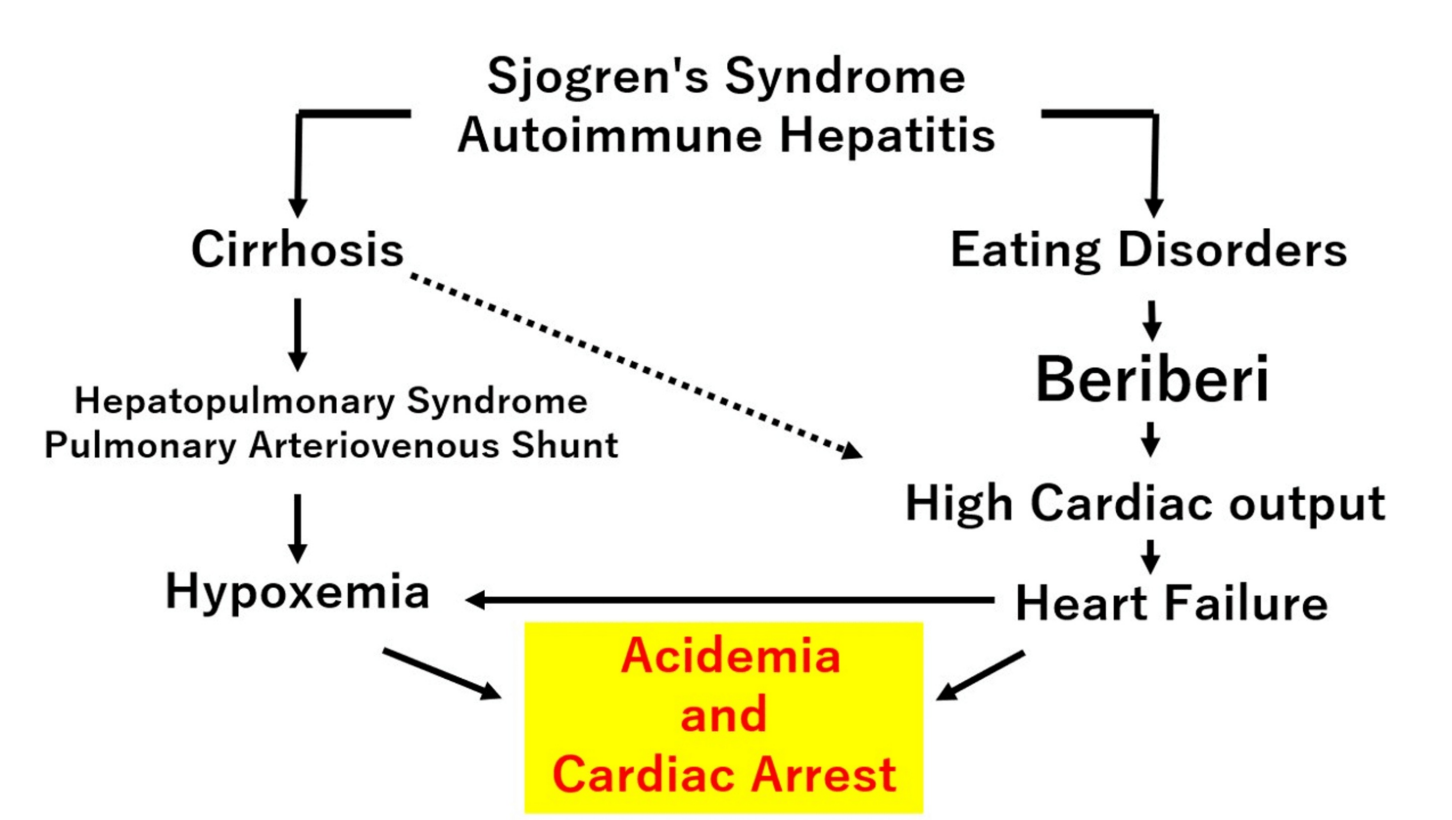
Pathophysiological mechanisms leading to hypoxemia and cardiac arrest in a patient with cirrhosis and beriberi heart disease Solid lines indicate causal relationships; dashed lines indicate possible associations. Image created by the author.

Postmortem external lab results confirmed low vitamin B1 levels, compatible with beriberi heart disease [11]. Because the patient never regained consciousness and the family had not previously discussed any symptoms with them, it remained unclear whether the patient had experienced any related complaints. However, high titers of antinuclear antibodies, SS-A antibodies, and anti-cyclic citrullinated peptide antibodies were detected, suggesting Sjögren's syndrome. These findings suggest that the cause of the cardiac arrest was considered to be the delivery of deoxygenated blood to the systemic circulation due to pulmonary arteriovenous shunts associated with cirrhosis caused by AIH and Sjögren's syndrome. This resulted in hypoxemia resistant to oxygen supplementation, inadequate systemic oxygen supply, resulting in acidemia, and ultimately caused cardiac arrest (Figure 4). While clubbing is known to be caused by chronic hypoxemia, it can also be associated with cirrhosis. The clubbing observed after resuscitation in this case was likely due to these combined factors. 

## Discussion

High-output heart failure and beriberi heart disease

High-output heart failure is associated with reduced SVR and increased metabolic demand [11]. Beriberi heart disease, caused by thiamine deficiency, is one such cause. Diagnosis relies on a history of malnutrition and hemodynamic improvement following thiamine administration [12,13]. In this case, preserved left ventricular ejection fraction along with elevated LVOT-VTI (left ventricular outflow tract velocity time integral), CO, and CI suggested a high-output state. Hemodynamic measurements via an SG catheter revealed that the mean PAWP exceeded the normal range of 8-15 mmHg, consistent with postcapillary pulmonary hypertension. PVR was significantly reduced compared to the normal upper limit of approximately 3 WU, while SVR remained near the lower end of the normal range (10-15 WU). CO and CI were markedly elevated beyond their respective normal ranges (4-8 L/minute and 2.2-4.0 L/minute/m^2^), supporting the presence of a high-output state [7].

These hemodynamic findings are characteristic of high-output heart failure, which is typically defined by increased CO due to reduced SVR, often in response to excessive metabolic demands or vasodilation. SG catheterization confirmed this diagnosis, and detailed history-taking combined with therapeutic response established beriberi as the underlying cause. Despite coexisting HPS, thiamine supplementation improved hemodynamics, indicating beriberi as the primary cause of high output.

Sjögren’s syndrome and autoimmune hepatitis

The postmortem examination revealed cirrhosis with lymphocytic infiltration, emperipolesis, bridging fibrosis, and destruction of normal lobular architecture. Emperipolesis is a characteristic finding in AIH and is more frequently seen in AIH than in other liver diseases [8]. Postmortem laboratory results suggested underlying Sjögren's syndrome. The coexistence of AIH and Sjögren's syndrome is well documented. Approximately 40% of AIH patients develop other autoimmune conditions, while AIH occurs in 4-47% of patients with Sjögren's syndrome [14,15].

Pathogenesis of hepatopulmonary syndrome

In cirrhosis and portosystemic shunting, gut-derived toxins and bacteria bypass the liver and enter systemic circulation, leading to bile duct proliferation, monocyte/macrophage activation, increased endothelin-1 production, and bacterial translocation, causing increased tumor necrosis factor α (TNFα) levels. TNFα also promotes vascular endothelial growth factor production, leading to pulmonary neovascularization. These mechanisms contribute to pulmonary vasodilation and the formation of pulmonary arteriovenous shunts, causing hypoxemia characteristic of HPS [2,3,16]. In this patient, undiagnosed Sjögren's syndrome likely led to AIH, culminating in cirrhosis and subsequent development of HPS.

Pulmonary arteriovenous shunts: diagnostic methods and limitations

A microbubble test using TTE is noninvasive and effective for detecting pulmonary arteriovenous shunts [4]. However, standard criteria may be unreliable in cases of hemodynamic instability, extremely high- or low-output states, or when intracardiac shunts cannot be excluded [5,6,17]. In addition, TEE may be contraindicated in patients with esophageal varices, which are common in cirrhosis. The coexistence of high-output states and HPS is not uncommon in advanced liver disease, necessitating careful consideration and tailored diagnostic approaches.

Microbubble testing using a Swan-Ganz catheter

In this case, characterized by the presence of beriberi heart disease and the unstable and unique hemodynamic conditions following resuscitation, as well as prolonged refractory hypoxemia, the performance of TEE posed significant risks. Consequently, direct injection of microbubbles into the pulmonary artery via an SG catheter was undertaken. Direct administration of microbubbles into the pulmonary artery through an SG catheter is considered a useful and effective method for providing definitive evidence of pulmonary arteriovenous shunting under such conditions.

When performing this test, it is crucial to ensure that the catheter tip is not in a wedged position or penetrating a branch vessel to avoid pulmonary artery or lung injury. Safety can be improved by using a pigtail catheter, conducting the procedure under fluoroscopic guidance, or verifying the tip pressure immediately prior to injection, as in this case. In the absence of prior reports, microbubbles were prepared following standard peripheral microbubble testing protocols, using a mixture of 8 mL saline, 1 mL air, and 1 mL patient blood [8]. However, considering the length of the infusion line and the rapid delivery of all administered microbubbles to the pulmonary artery, it may be possible to achieve diagnosis with a smaller volume.

Although no previous cases have employed this method to diagnose pulmonary arteriovenous shunts, its reliance on commonly used cardiovascular equipment and techniques underscores its potential as a valuable diagnostic approach. For future clinical applications, further studies are warranted to identify the optimal volume of microbubble mixture required to achieve sufficient diagnostic yield while minimizing the risk of pulmonary complications. However, given the rarity of conditions necessitating this approach, prospective studies may be difficult to conduct. Instead, the accumulation of case-based experience and the sharing of findings through case reports or registries would be desirable to refine safety parameters, optimize protocols, and establish this method as a practical diagnostic tool in complex clinical scenarios.

## Conclusions

This case suggests that cirrhosis resulting from AIH and subsequent HPS led to refractory hypoxemia and, ultimately, cardiac arrest. Administering microbubbles directly into the pulmonary artery through an SG catheter allowed for a rapid and minimally invasive approach to diagnosing a pulmonary arteriovenous shunt. This method may be particularly useful in cases with complex hemodynamics or anatomical considerations that limit conventional diagnostic approaches. Because it employs commonly available cardiovascular equipment, this approach could be widely implemented, enhancing diagnostic accuracy and informing treatment strategies in similar clinical scenarios. Given the rarity of conditions requiring such a diagnostic approach, the accumulation of case-based experience through reports and registries would help refine safety parameters and optimize protocols, ultimately establishing this method as a practical diagnostic tool for complex clinical scenarios.
